# A retrospective analysis of lymph node dissection in Siewert II adenocarcinoma of the esophagogastric junction

**DOI:** 10.1186/s13019-024-02897-3

**Published:** 2024-07-19

**Authors:** Yang Tian, Hiulai Lv, Mingbo Wang, Ziqiang Tian

**Affiliations:** 1 Department of Thoracic and Cardiac Surgery, Hangzhou Hospital of Traditional Chinese Medicine, Hangzhou, China; 2 Department of Thoracic Surgery, The Fourth Hospital of Hebei Medical University, NO. 12, JianKang Road, Shijiazhuang, China; 3 Hebei Provincial Key Laboratory of Precision Diagnosis and Comprehensive Treatment of Esophageal Cancer, Hebei, China

**Keywords:** Adenocarcinoma of the esophagogastric junction, Lymph node dissection, Ivor-Lewis

## Abstract

**Background:**

Analyze the pattern of lymph node metastasis in Siewert II adenocarcinoma of the esophagogastric junction (AEG) and provide a basis for the principles of surgical access.

**Methods:**

The clinical data of 112 Siewert type II AEG patients admitted to the Fifth Department of Thoracic Surgery, the Fourth Hospital of Hebei Medical University from 2020 to 2022 were retrospectively collected. The probability of lymph node metastasis in each site and the clearance rate of lymph nodes in each site by different surgical approaches were analyzed.

**Results:**

The lymph node metastasis rates in the middle and upper mediastinum group, the lower mediastinum group, the upper perigastric + supra pancreatic group, and the lower perigastric + hepatoduodenal group were 0.0%, 5.4%, 61.6%, and 17.1%, (*P* < 0.001). The number of lymph nodes cleared in the middle and upper mediastinum group was 0.00, 0.00, 4.00 in the transabdominal approach (TA), left thoracic approach (LT), and Ivor-Lewis (IL) group, (*P* < 0.001); The number of lymph nodes cleared in the lower mediastinal group was 0.00, 2.00, 2.00, (*P* < 0.001); The number of lymph node dissection in the perigastric + hepatoduodenal group was 3.00, 0.00, and 8.00, (*P* < 0.001). The overall complication rates were 25.7%, 12.5%, and 36.4%, (*P* = 0.058).

**Conclusion:**

Siewert II AEG has the highest rate of lymph node metastasis in the upper perigastric + supra-pancreatic region, followed by the lower perigastric + hepatoduodenal, lower mediastinal, middle, and upper mediastinal regions. Ivor-Lewis can be used for both thoracic and abdominal lymph node dissection and does not increase the incidence of postoperative complications.

In recent years, the incidence of adenocarcinoma of the esophagogastric junction (AEG) has been rapidly increasing worldwide [1], causing widespread concern in the medical field. Due to the high malignancy of AEG, lack of early symptoms, and low coverage of endoscopic examination for gastrointestinal tumors, some patients are already in the middle and late stages upon hospital arrival, with poor treatment effects and low five-year survival rates [2]. Siewert AEG has a special onset location, complex lymph node metastasis pathway, and controversial lymph node clearance scope [3]. This study included the clinical data of 112 patients who underwent surgical treatment of Siewert II AEG in our department from January 2020 to January 2022 and analyzed the pattern of lymph node metastasis, aiming to provide a rational basis for selecting surgical access for patients with Siewert II AEG.

## Materials and methods

### Clinical information

This study retrospectively collected clinical data of patients who underwent surgical resection of Siewert II AEG at the Fourth Hospital of Hebei Medical University from January 2020 to January 2022. Inclusion criteria: (1) gastroscopy and pathologically confirmed Siewert II AEG, (2) no combined history of other malignancies, (3) no combined history of other thoracic or abdominal surgeries, and (4) complete clinical data. A total of 112 patients were included in this study according to the inclusion criteria, and the specific clinical characteristics are shown in Table 1.

**Table 1:** Clinical characteristics of 112 patients with esophageal-gastric junction cancer

Clinical Features	TA Group (*n* = 39)	LT Group (*n* = 40)	IL Group (*n* = 33)	*P*
Gender				0.325
Male Female	29 10	35 5	27 6	
Age				0.084
≤60 >60	16 23	10 30	6 27	
Neoadjuvant therapy				0.765
Yes No	17 22	15 25	15 18	
ASA				0.679
1 2 3 4	19 18 2 0	24 15 1 0	20 13 0 0	
R0 resection				0.643
Yes No	38 1	40 0	33 0	
Tumor diameter				0.789
≤4 cm >4 cm	22 17	21 19	20 13	
Degree of differentiation				0.141
High differentiation Middle Divergence Low differentiation	5 16 18	7 21 12	3 10 20	
pTNM staging				0.155
I II III	9 11 19	11 18 11	4 12 17	

### Surgery method

The clinical data of 112 patients who underwent Siewert II AEG surgery from January 2020 to January 2022 in a single surgical group in our department were included in this study and were divided into transabdominal access group, left thoracic access group, and Ivor-Lewis group according to the surgical approach.

TA group: The esophagus was cut off about 5 cm proximal to the tumor, and the gastric body was cut off 5 cm distal to the tumor. An end-to-side anastomosis of the remnant esophagus and stomach was performed, and the remnant stomach wall was suspended by intermittent suture with the diaphragm.

TT group: The esophagus was transected under the aortic arch, and the gastric body was transected at a distance of 5 cm distal to the tumor. An end-to-side anastomosis of the remnant esophagus and stomach was performed, and the remnant stomach wall was suspended by intermittent suture with the diaphragm.

IL group: We cover the azygos vein severed esophagus, stomach making tubular stomach, pull from the diaphragmatic hiatus tubular stomach, esophagus stomach end side anastomosis.

### Lymph node grouping

**Fig. 1:**
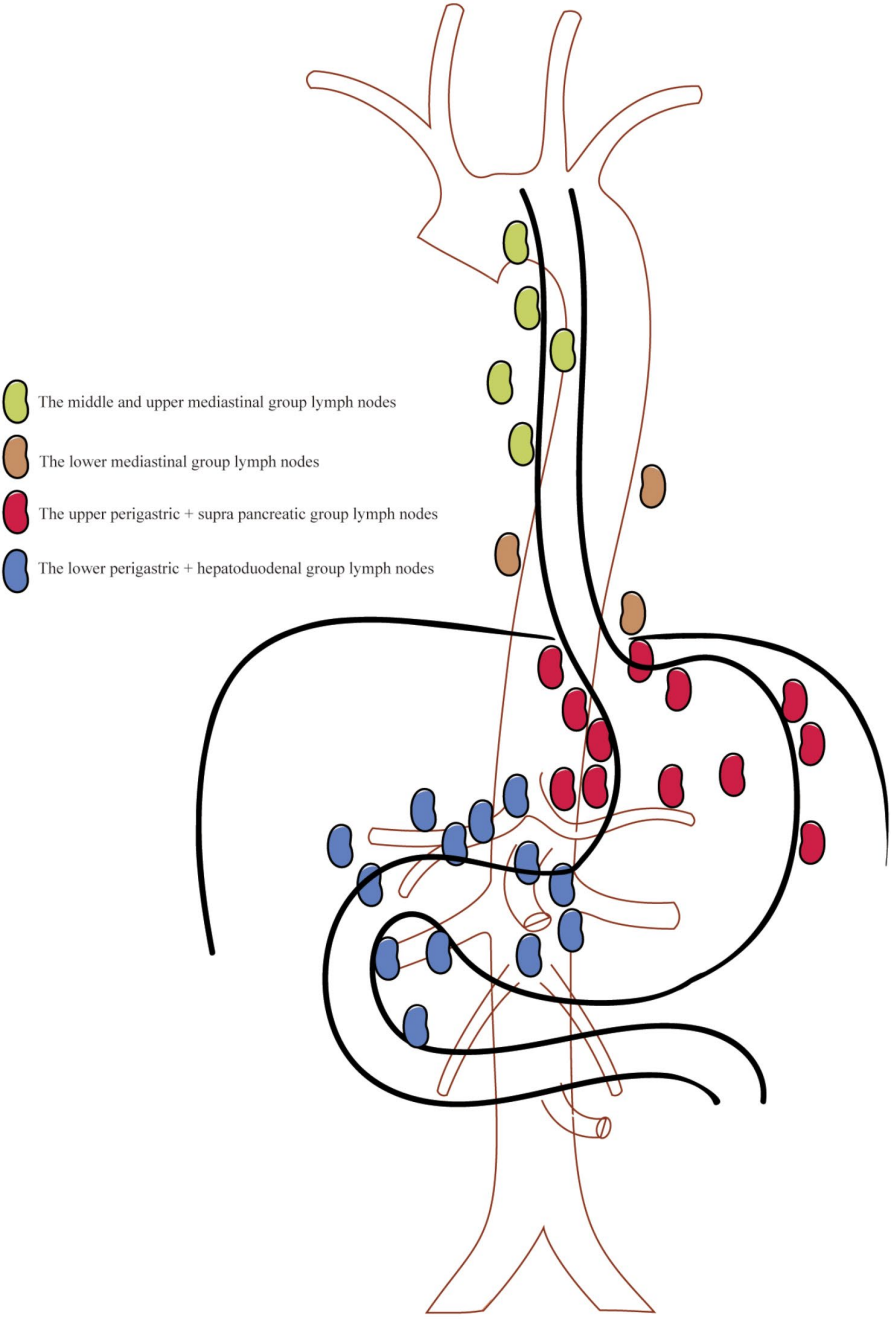
Schematic representation of lymph node grouping

According to the regional lymph node grouping criteria of the Japanese Esophageal Association, the thoracoabdominal-associated lymph nodes were divided into four groups according to their anatomical location and the ease of resection by different surgical approaches in this study: the middle and upper mediastinal group, the lower mediastinal group, the upper perigastric + supra pancreatic group, and the lower perigastric + hepatoduodenal group. (1) Lymph nodes of the middle and upper mediastinal groups: the group of lymph nodes more favorable for complete resection through the right thoracic approach, including the 106recL group, 106recR group, 106pre group, 105 group, 108 group, 107 group, and 109 group. (2) Lower mediastinal cluster lymph nodes: the transthoracic approach is more favorable for the complete lymph node resection group, including the 110 group, 112aoA group, 112aoP group, 111 group, and 112pul group. (3) Upper perigastric + supra pancreatic group lymph nodes: the main part of lymph node resection for Siewert II adenocarcinoma of the esophagogastric junction can be more completely resected by either transthoracic or transabdominal approach, including groups 1, 2, 3, 3a, 4sa, 4sb, 7, 10, 11p, 11d, 19, 20. (4) Lower perigastric + hepatoduodenal group lymph nodes: the transabdominal approach is more favorable for complete resection of the lymph node groups, including group 3b, group 4d, group 5, group 6, group 8a, group 8p, group 9, group 12, group 13, group 14, group 15, group 16, group 17. Specific schematic representation of lymph node grouping shown in Fig. 1.

### Statistical methods

The enrolled data were statistically analyzed using SPSS 23, the median (quartiles) was used for measurement data, and the Kruskal-Wallis H test was used for comparison between groups; the count data were expressed as percentages, and the chi-square test or Fisher’s exact test was used for comparison between groups, with *P* < 0.05 indicating a statistically significant difference.

## Results

### Lymph node metastasis rate and metastasis degree of each group

The rates of lymph node metastasis in the middle and upper mediastinal groups, lower mediastinal group, upper perigastric + supra pancreatic group, and lower perigastric + hepatoduodenal group were 0.0%, 5.4%, 61.6%, and 17.1%, respectively, with statistically significant differences (*P* < 0.001). The rate of lymph node metastasis in the upper perigastric + supra pancreatic group was significantly higher than that in the lower perigastric + hepatoduodenal group (*P* < 0.001). duodenal group lymph node metastasis rate was significantly higher than that of the middle and upper mediastinal group and the lower mediastinal group (*P* < 0.05), and the difference between the lymph node metastasis rate of the middle and upper mediastinal group and the lower mediastinal group was not statistically significant (*P* = 0.325). The lymph node metastases were 0.0%,4.8%,27.0%, and 7.0%, respectively. The differences were statistically significant (*P* < 0.001). The lymph node metastases in the upper perigastric + supra pancreatic group were significantly higher than those in the lower mediastinal group. The lower perigastric + hepatoduodenal group (*P* < 0.001), and the lymph node metastases in the lower mediastinal group and the lower perigastric + hepatoduodenal group were both significantly higher than those in the middle and upper mediastinal groups (*P* < 0.05). In contrast, the difference in lymph node metastasis between the inferior mediastinal group and the inferior perigastric + hepatoduodenal group was not statistically significant (*P* = 0.287). Table 2.

**Table 2:** Comparison of lymph node metastasis rate, metastasis degree in each group

Lymph node groups	Number of positive cases (cases)	Number of cleaning cases (cases)	Lymph node metastasis rate (%)	Number of positive lymph nodes(pcs)	Number of lymph nodes cleared(pcs)	Lymph node metastasis degree(%)
Upper middle mediastinum	0	33	0.0	0	141	0.0
inferior mediastinum	5	93	5.4	10	207	4.8
Perigastric + Suprapancreatic	69	112	61.6	401	1485	27.0
Perigastric + hepatoduodenal	13	76	17.1	32	457	7.0
P-value	-	-	< 0.001	-	-	< 0.001

**Lymph node metastasis rate**: mid-upper mediastinal cluster vs. lower mediastinal cluster, *P* = 0.325; mid-upper mediastinal cluster vs. perigastric + supra pancreatic cluster, *P* < 0.001; mid-upper mediastinal cluster vs. lower perigastric + hepatoduodenal cluster, *P* = 0.009; lower mediastinal cluster vs. perigastric + supra pancreatic cluster, *P* < 0.001; lower mediastinal cluster vs. lower perigastric + hepatoduodenal cluster, *P* = 0.014; upper perigastric + supra pancreatic cluster vs. inferior perigastric + hepatoduodenal cluster, *P* < 0.001

**Degree of lymph node metastasis**: mid-upper mediastinal cluster vs. lower mediastinal cluster, *P* = 0.007; mid-upper mediastinal cluster vs. upper perigastric + supra pancreatic cluster, *P* < 0.001; mid-upper mediastinal cluster vs. lower perigastric + hepatoduodenal cluster, *P* = 0.001; lower mediastinal cluster vs. upper perigastric + supra pancreatic cluster, *P* < 0.001; lower mediastinal cluster vs. lower perigastric + hepatoduodenal cluster *P* = 0.287; upper perigastric + supra pancreatic cluster vs. inferior perigastric + hepatoduodenal cluster, *P* < 0.001

### Lymph node metastasis rate of each group in different pTNM stages

The lymph node metastasis rates in the upper mediastinal group were 0.0% in the stage I group, stage II group, and stage III group; the lymph node metastasis rates in the lower mediastinal group were 0.0%, 3.6%, and 14.8%, respectively, with no statistically significant difference (*P* = 0.355); the lymph node metastasis rates in the upper perigastric + supra pancreatic group were 4.5%, 46.3%, and 100%, respectively, with statistically significant differences (*P* < 0.001). The lymph node metastasis rates in the stage III group were significantly higher than those in the stage II group (*P* < 0.001). The lymph node metastasis rate of the upper perigastric + supra pancreatic group was significantly higher than that of the stage II group (*P* < 0.001), and that of the stage II group was significantly higher than that of stage I group (*P* = 0.001); the lymph node metastasis rates of lower perigastric + hepatoduodenal group were 0.0%, 3.8%, and 30%, and the differences were statistically significant (*P* = 0.008), and that of lower perigastric + hepatoduodenal group was significantly higher than that of stage II group in stage III group (*P* = 0.009), and the rate of lymph node metastasis in the lower perigastric + hepatoduodenal group in the stage II group was not statistically significantly different from that in the stage I group (*P* = 1.000). Table 3.

**Table 3:** Comparative results of lymph node metastasis in different pTNM stages and different partitions

Lymph node groups	Stage I group			Stage II group			Stage III group			*P*-value
	Number of positive cases (cases)	Number of cleaning cases (cases)	Lymph node metastasis rate (%)	Number of positive cases (cases)	Number of cleaning cases (cases)	Lymph node metastasis rate (%)	Number of positive cases (cases)	Number of cleaning cases (cases)	Lymph node metastasis rate (%)	
Upper middle mediastinum	0	2	0.0	0	12	0.0	0	16	0.0	
inferior mediastinum	0	10	0.0	0	28	3.6	4	27	14.8	0.355
Perigastric + Suprapancreatic	1	22	4.5	19	41	46.3	49	49	100.0	< 0.001
Perigastric + hepatoduodenal	0	10	0.0	1	26	3.8	12	40	30.0	0.008

**Upper perigastric + supra pancreatic group lymph node metastasis rate: stage** I group vs. stage II group, *P* = 0.001; stage I group vs. stage III group, *P* < 0.001; stage II group vs. stage III group, *P* < 0.001

**Perigastric + hepatoduodenal group lymph node metastasis rate: stage** I group vs. stage II group, *P* = 1.000; stage I group vs. stage III group, *P* = 0.092; stage II group vs. stage III group, *P* = 0.009

### The number of lymph node dissections in each group in different surgery groups

The number of lymph nodes cleared in the upper mediastinal group was 0.00 (0.00–0.00), 0.00 (0.00–0.00), 4.00 (2.00–6.00) in the TA, LT, and IL groups, and the difference was statistically significant (*P* < 0.001), and the number of lymph nodes cleared in the upper mediastinal group was significantly higher in the IL group than in the TA and LT groups (*P* < 0.001). The number of lymph nodes cleared in the upper mediastinal group in the TA and LT groups was not statistically significantly different (*P* = 1.000); the number of lymph nodes cleared in the lower mediastinal group was 0.00 (0.00–0.00), 2.00 (1.00–3.00), 2.00 (1.00–4.00), and the difference was statistically significant (*P* < 0.001), and the number of lymph nodes cleared in the lower mediastinal group in the LT and IL groups was significantly higher than that in the TA group. The number of lymph nodes cleared in the lower mediastinal group was significantly higher in both the LT and IL groups than in the TA group (*P* < 0.001), and there was no statistically significant difference in the number of lymph nodes cleared in the lower mediastinal group between the LT and IL groups (*P* = 1.000); the number of lymph nodes cleared in the upper perigastric + suprapancreatic group was 11.00 (7.00–16.00), 10.50 (7.25-18.00), and 12.00 (10.00-22.50), with no statistically significant difference There was no statistically significant difference (*P* = 0.055); the number of lymph nodes cleared in the lower perigastric + hepatoduodenal group was 3.00 (1.00–7.00), 0.00 (0.00–1.00), 8.00 (4.00–12.00), and the difference was statistically significant (*P* < 0.001), and the number of lymph nodes cleared in the lower perigastric + hepatoduodenal group in both TA and IL groups was significantly The number of lymph node dissection in the lower perigastric + hepatoduodenal group was significantly higher in the TA and IL groups than in the LT group (*P* < 0.001). Table 4.

**Table 4:** Comparison of the number of lymph node dissections in different operation groups and different partitions [M(Q1-Q3)]

Lymph node group	TA Group	LT Group	IL Group	*P*-value
Upper middle mediastinum	0.00 (0.00–0.00)	0.00 (0.00–0.00)	4.00 (2.00–6.00)	< 0.001
inferior mediastinum	0.00 (0.00–0.00)	2.00 (1.00–3.00)	2.00 (1.00–4.00)	< 0.001
Perigastric + Suprapancreatic	11.00 (7.00–16.00)	10.50 (7.25-18.00)	12.00 (10.00-22.50)	0.055
Perigastric + hepatoduodenal	3.00 (1.00–7.00)	0.00 (0.00–1.00)	8.00 (4.00–12.00)	< 0.001

**The number of lymph nodes cleared in the middle and upper mediastinal area**: TA group vs. LT group, *P* = 1.000; TA group vs. IL group, *P* < 0.001; LT group vs. IL group, *P* < 0.001

**Number of lymph nodes cleared in the inferior mediastinal region**: TA group vs. LT group, *P* < 0.001; TA group vs. IL group, *P* < 0.001; LT group vs. IL group, *P* = 1.000

**The number of lymph nodes cleared** in the **perigastric + hepatoduodenal region**: TA group vs. LT group, *P* < 0.001; TA group vs. IL group, *P* = 0.005; LT group vs. IL group, *P* < 0.001

### Operative time, intraoperative bleeding, and overall complication rate of each operation group

The operative times in the TA, LT, and IL groups were 208 (175–222) min, 169 (150–183) min, and 289.00 (259.00-339.50 ) min, with statistically significant differences (*P* < 0.001), and the operative times in the IL group were significantly longer than those in the TA group, and in the TA group was significantly longer than those in the LT group (*P* < 0.05); intraoperative bleeding was 120 (100–200), 150 (100–200) and 200.00 (150.00-250.00), with statistically significant differences (*P* < 0.001), and intraoperative bleeding was significantly more in the IL group than in the LT and IL groups (*P* < 0.05), and there was no statistically significant intraoperative bleeding in the LT and IL groups There was no statistically significant difference between the LT and IL groups (*P* = 0.665). The overall complication rates were 25.7%, 12.5%, and 36.4%, with no statistically significant differences (*P* = 0.058). Tables 5 and 6.

**Table 5:** Comparison of operative time and intraoperative bleeding in each surgical group [M(Q1-Q3)

Comparative indicators	TA Group	LT Group	IL Group	*P*-value
Surgery time(min)	208(175–222)	169(150–183)	289.00 (259.00-339.50)	< 0.001
Intraoperative bleeding (ml)	120(100–200)	150(100–200)	200.00 (150.00–250.00)	< 0.001

**Operative time**: TA group vs. LT group, *P* = 0.001; TA group vs. IL group, *P* < 0.001; LT group vs. IL group, *P* < 0.001

**Intraoperative bleeding**: TA group vs. LT group, *P* = 0.665; TA group vs. IL group, *P* < 0.001; LT group vs. IL group, *P* = 0.001

**Table 6:** Incidence of complications in each surgical group. N(%)

Comparative Metrics	TA group (*n* = 39)	LT group (*n* = 40)	IL group (*n* = 33)	*P*-value
Pneumonia	7(17.9)	4(10.0)	4(12.1)	
Arrhythmia	4(10.3)	3(7.5)	5(15.2)	
Celiac disease	0(0.0)	0(0.0)	1(3.0)	
Respiratory failure	3(7.7)	0(0.0)	3(9.1)	
Anastomotic fistula	1(2.6)	1(2.5)	2(6.1)	
Unplanned secondary surgery	1(2.6)	0(0.0)	1(3.0)	
Complication-related deaths	1(2.6)	0(0.0)	2(6.1)	
Total complication rate	10(25.7)	5(12.5)	12(36.4)	0.058

## Discussions

According to the widely accepted Siewert’s staging, cancers with a tumor center located within 5 cm proximal and distal to the EGJ are called esophagogastric junction cancers, and are classified into three types according to the distance of the tumor center from the esophagogastric junction (EGJ): Type I with the tumor center located 1–5 cm above the EGJ; II with the tumor center located 1–2 cm below the EGJ; III with the tumor center located 2–5 cm below the EGJ. Type I is tumor-centered 1–5 cm above EGJ; II is tumor-centered 1–2 cm below EGJ; III is tumor-centered 2–5 cm below EGJ [4–5]. The incidence ratio of the three subtypes is approximately the same in Western countries, while in China, Korea, Japan, and other Eastern countries, Siewert II and Siewert III account for the majority of the three subtypes, while Siewert I accounts for a smaller proportion [6–7]. According to statistics, the incidence of esophagogastric junction cancer has been rapidly increasing worldwide in recent years [8], and its five-year survival rate hovers at only 20% in many groups of the world [9]. However, since the cancer is located at the junction of the esophagus and stomach, its lymph nodes can metastasize upward to mediastinal lymph nodes and downward to abdominal lymph nodes, it is especially important to master the rule of lymph node metastasis and determine the reasonable surgical access and lymph node dissection range for the long-term survival rate of patients. We included 112 patients with Siewert II adenocarcinoma of the esophagogastric junction who underwent single-incision surgery in the upper abdomen, single-incision surgery in the left chest, and Ivor-Lewis surgery. The aim is to provide a reasonable basis for the selection of surgical treatment for Siewert II esophagogastric adenocarcinoma.

### Metastasis rate and degree of metastasis in each group of lymph nodes

A prospective multicenter study by Kurokawa [10] et al. showed that for squamous and adenocarcinoma of cT2-T4 within 2 cm of the EGJ, the lymph node metastasis rate in groups 1, 2, 3, 7, 9, and 11p was more than 10% and strongly recommended for clearance, and the lymph node metastasis rate in groups 8a, 19, and 110 was between 5% and 10% and weakly recommended for clearance; if esophageal involvement was more than 2 cm, the lymph node metastasis rate in groups 106recR and 110 was 10.8% and strongly recommended for clearance, The metastatic rate of other mediastinal lymph nodes was less than 5%. Subgroup analysis of the included cases showed that when the esophageal invasion was more than 2.0 cm, only 110 groups of mediastinal lymph nodes were upgraded to type 1 lymph nodes, and the metastasis rate of other mediastinal lymph nodes was also less than 5%. When the esophageal invasion was more than 3.0 cm, the mediastinal lymph node metastasis rate was 105 (0.0%), 106recR (2.9%), 106recL (0.0%), 107 (1.4%), 108 (4.3%), 109R (1.4%), 109 L (1.4%), 110 (20.8%). 111 (3.8%), 112 (1.9%); When the esophageal invasion was more than 4.0 cm, the mediastinal lymph node metastasis rate was 105 (3.6%), 106recR (10.7%), 106recL (3.6%), 107 (7.1%), 108 (7.1%), 109R (3.6%), 109 L (7.1%), 110 (28.6%). 111 (10.7%), 112 (7.1%). A retrospective study of 395 cases of Siewert II and III combined esophagogastric carcinoma A retrospective study of 395 Siewert II and III combined esophagogastric cancers showed that the lymph node metastasis rate in groups 1, 2, 3, and 7 exceeded 20%, and the lymph node metastasis rate in groups 9 and 11p exceeded 10%, and the index of estimated benefit from lymph node dissection IEBLD in these groups was > 3.0, which could have a high therapeutic benefit for patients. The estimated benefit from lymph node dissection in these groups (index of estimated benefit from lymph node dissection IEBLD) was > 3.0, which could produce high treatment benefits for patients [11]. The results of this study showed that the lymph node metastasis rate and degree of metastasis in the supra gastric + supra pancreatic group were significantly higher than those in the other three groups, the lymph node metastasis rate in the hypogastric + hepatoduodenal group was significantly higher than that in the inferior mediastinal group, and no lymph node metastasis was seen in the middle and superior mediastinal group, which is more consistent with the results of the above-mentioned study on lymph node metastasis rate in each group [13, 14]. The combination of thoracic lymph node dissection with adequate lymph node dissection in the abdominal cavity may be significant for the long-term survival of patients. In conclusion, for most patients with Siewert II esophagogastric junction adenocarcinoma, when the tumor invasion is not more than 4 cm, upper and middle mediastinal lymph node dissection may not bring survival benefits while increasing surgical trauma. Furthermore, is abdominal + left thoracic approach feasible when upper and middle mediastinal lymph node dissection is optional? From the anatomical point of view, the lower segment of the esophagus is more inclined to the left side, and the position of the left diaphragm is lower than that of the right side. These natural anatomical advantages make the left thoracic approach have certain advantages in terms of mobilization and anastomosis. Of course, these conjectures need to be further verified.

### Lymph node metastasis rate at different pTNM stages

The results of this study showed that along with the tumor progression, the metastasis rate of lymph nodes in the middle and upper mediastinal groups was 0.0%, while the metastasis rate of lymph nodes in the lower mediastinal group had a tendency to increase but there was no significant statistical difference, and the metastasis rate of lymph nodes in the upper perigastric + supra pancreatic group and the lower perigastric + hepatoduodenal group were both significantly higher. This indicates that the more advanced the tumor stage is, the more prominent the value of abdominal lymph nodes (upper perigastric + supra pancreatic and lower perigastric + hepatoduodenal lymph nodes) than thoracic lymph nodes (middle and upper mediastinum and lower mediastinum lymph nodes) is, and this result has guiding significance for the selection of clinical surgery.

### Number of lymphatic dissections in each group in different surgical groups

The results of this study showed that the number of lymph nodes cleared in the upper and middle mediastinal groups was significantly higher in the IL group than in the LT and TA groups, the number of lymph nodes cleared in the lower mediastinal group was significantly higher in both the IL and LT groups than in the TA group, and the number of lymph nodes cleared in the lower perigastric + hepatoduodenal group was significantly higher in both the IL and TA groups than in the LT group, and there was no statistically significant difference between the lymph cleared in the upper perigastric + supra pancreatic group in each surgical group. This demonstrates that the Ivor-Lewis procedure has significant advantages over other single-incision procedures in terms of lymph node clearance, and can take into account the adequate clearance of lymph nodes in the middle and upper mediastinal groups, the lower mediastinal group, the upper perigastric + supra pancreatic group, and the lower perigastric + hepatoduodenal group, which may be significant for the long-term survival of patients.

### Operative time, intraoperative bleeding, and overall complication rate of each operation group

A comparative study of surgical approaches in 331 patients with SIEWERT II AEG by Zheng [12] et al. showed no significant differences in operative time, intraoperative bleeding, overall complication rate, postoperative pneumonia, pleural effusion, incisional infection, gastrointestinal dysfunction, anastomotic fistula, and unplanned secondary surgery between the transthoracic approach and transabdominal approach groups. A single-center study by Yang [13] et al. suggested that the transthoracic approach had a longer operative time and a higher incidence of postoperative complications (Clavien-Dindo classification ≥ grade II) than the transabdominal approach, but there was no significant difference in 30-day mortality between the two groups. Duan [14] et al. showed no significant difference in intraoperative bleeding and postoperative complication rates between Ivor-Lewis and transabdominal approach, but longer operative time for Ivor-Lewis. A single-center study by Blank [15] et al. A single-center study by Blank [16] et al. showed no significant differences in total postoperative complication rates, anastomotic fistula, pulmonary and cardiovascular complications, serious complications (Clavien-Dindo > IIIA), and 30-day mortality between the Ivor-Lewis and transabdominal approach groups. However, there was no significant difference in the incidence of pulmonary and cardiovascular complications, serious complications (Clavien-Dindo > IIIA), 30-day mortality, or 90-day mortality. The results of this study showed that the Ivor-Lewis group had a significantly longer operative time than the TA and LT groups, and significantly more intraoperative bleeding than the LT and TA groups, but did not increase the incidence of postoperative complications and complication-related mortality rates in a way that is not entirely consistent with the above-mentioned studies, which may be due to differences in surgical proficiency and perioperative management among surgeons at each medical institution, but all of the above-mentioned studies showed that Ivor-Lewis at least did not increase the incidence of serious complications (Clavien-Dindo > IIIA). Lewis at least did not increase the incidence of serious complications (Clavien-Dindo > IIIA), demonstrating the feasibility of the IL procedure, However, due to the limited sample size and absence of quality-of-life data about postoperative reflux esophagitis, loss of body mass index, and severity of hoarseness in each surgical group, further analysis regarding the aggressiveness of the Ivor-Lewis procedure could not be conducted. So a careful and progressive surgical plan based on the patient’s clinical stage, physical condition, and level of care at the institution is still needed.

In conclusion, for Siewert II esophagogastric junction adenocarcinoma, the highest rate of lymph node metastasis was observed in the upper perigastric + supra pancreatic group, followed by the lower perigastric + hepatoduodenal group, and the lower mediastinal group. Ivor-Lewis procedure has significant advantages in terms of lymph node clearance. The Ivor-Lewis procedure has obvious advantages in lymph node dissection and can adequately dissect the lymph nodes in the middle and upper mediastinal groups, the lower mediastinal group, the upper perigastric + supra pancreatic group, and the lower perigastric + hepatoduodenal group, and does not increase the postoperative complication rate, which proves that the Ivor-Lewis procedure is feasible, but since this procedure involves many disciplines and the perioperative complications have increased in some medical institutions, it is still necessary to choose carefully according to the actual patient condition and clinical stage. However, since this procedure involves many disciplines and perioperative complications in some medical institutions, it should be selected carefully and carried out gradually according to the patient’s condition and clinical stage. In addition, this study is a single-center small sample study with certain limitations, and the lymph node metastasis rate and the number of cleared lymph nodes may be biased by the artificial selection of different surgical procedures, and further studies are needed to compare the long-term survival rate of patients to obtain more valuable statistical results.

## Data Availability

No datasets were generated or analysed during the current study.

## References

[1] Nishiwaki N, Noma K, Matsuda T, et al. Risk factor of mediastinal lymph node metastasis of Siewert type I and II esophagogastric junction carcinomas [J]. Langenbecks Arch Surg. 2020;405(8):1101–9. 33155069 10.1007/s00423-020-02017-4

[2] Su G, Han Y, Bai YX. Research progress of immunotherapy for gastric and esophagogastric union cancer[J]. China Oncol. 2019;28(08):615–20.

[3] Munitiz V, Ortiz A, Ruiz de Angulo D, et al. Results of the different surgical options for the treatment of cancer of the esophagogastric junction: review of the evidence [J]. Cir Esp (Engl Ed). 2019;97(8):445–50. 10.1016/j.ciresp.2019.03.005 31027834

[4] Siewert JR, Hölscher AH, Becker K et al. Cardia cancer: attempt at a therapeutically relevant classification [J]. Chirurg. 1987;58(1):25–32. 3829805

[5] Siewert JR, Stein HJ. Classification of adenocarcinoma of the oesophagogastric junction [J]. Br J Surg. 1998;85(11):1457–9. 9823902 10.1046/j.1365-2168.1998.00940.x

[6] Giacopuzzi S, Bencivenga M, Weindelmayer J, et al. Western strategy for EGJ carcinoma [J]. Gastric Cancer. 2017;20(Suppl 1):60–8. 28039533 10.1007/s10120-016-0685-2

[7] Zhang H, Wang W, Cheng Y, et al. Adenocarcinomas of the esophagogastric junction: experiences at a single institution in China [J]. World J Surg Oncol. 2013;11:155. 23849250 10.1186/1477-7819-11-155 PMC3720177

[8] Chevallay M, Bollschweiler E, Chandramohan SM, et al. Cancer of the gastroesophageal junction: a diagnosis, classification, and management review [J]. Ann N Y Acad Sci. 2018;1434(1):132–8. 30138540 10.1111/nyas.13954

[9] Lorenzen S, Knorrenschild JR, Pauligk C, et al. Stage III randomized, double-blind study of paclitaxel with and without everolimus in patients with advanced gastric or esophagogastric junction carcinoma who have progressed after therapy with a fluoropyrimidine/platinum-containing regimen (RADPAC) [J]. Int J Cancer. 2020;147(9):2493–502. 32339253 10.1002/ijc.33025

[10] Kurokawa Y, Takeuchi H, Doki Y, et al. Mapping of Lymph Node Metastasis from Esophagogastric Junction Tumors [J]. Ann Surg. 2020 [Online ahead of print].

[11] Cai MZ, Lv CB, Cai LS, et al. Priority of lymph node dissection for advanced esophagogastric junction adenocarcinoma with the tumor center located below the esophagogastric junction [J]. Med (Baltim). 2019;98(51):e18451. 10.1097/MD.0000000000018451 PMC6940055 31861019

[12] Zheng B, Chen YB, Hu T, et al. Comparison of transthoracic and transabdominal surgical approaches for the treatment of adenocarcinoma of the cardia [J]. Chin J Cancer. 2010;29(8):747–51. 20663322 10.5732/cjc.009.10748

[13] Yang ZF, Wu DQ, Wang JJ, et al. Surgical approach for Siewert II adenocarcinoma of the esophagogastric junction: transthoracic or transabdominal? - a single-center retrospective study [J]. Ann Transl Med. 2018;6(23):450. 30603638 10.21037/atm.2018.10.66 PMC6312814

[14] Duan XF, Shang XB, Tang P, et al. Lymph node dissection for Siewert II esophagogastric junction adenocarcinoma [J]. ANZ J Surg. 2018;88(4):264–7. 28503799 10.1111/ans.13980

[15] Blank S, Schmidt T, Heger P, et al. Surgical strategies in true adenocarcinoma of the esophagogastric junction (AEG II). Thoracoabdominal or abdominal approach? [J]. Gastric Cancer. 2018;21:303–14. 28685209 10.1007/s10120-017-0746-1

[16] Tosolini C, Reim D, Schirren R, et al. Influence of the surgical technique on survival in the treatment of carcinomas of the true cardia (Siewert II)- right Thoracoabdominal vs transhiatal-abdominal approach [J]. Eur J Surg Oncol. 2019;45(3):416–24. 30396809 10.1016/j.ejso.2018.09.017

